# Modified EXTREME regimen versus modified TPEx regimen as first-line treatment for recurrent or metastatic head and neck squamous cell carcinoma: a multicenter, open-label, randomized, exploratory study (TEMPER study)

**DOI:** 10.1007/s10147-026-02964-w

**Published:** 2026-02-09

**Authors:** Motoyuki Suzuki, Akehito Kishino, Masashi Sugasawa, Takashi Fujii, Ayako Nakanome, Tomonori Terada, Hitoshi Hirakawa, Yushi Ueki, Mioko Matsuo, Rie Ito, Muneki Hotomi, Masafumi Kanno, Akihito Watanabe, Yoshiaki Kitamura, Kenji Okami, Kazuhira Endo, Takeharu Ono, Shigeru Hirano, Go Omura, Koichi Omori, Yoshifumi Yamamoto, Mototsugu Shimokawa, Hidenori Inohara

**Affiliations:** 1 Department of Otorhinolaryngology-Head and Neck Surgery, The University of Osaka, 2-2 Yamadaoka, Suita, Osaka 565-0871 Japan; 2 Department of Otolaryngology, Faculty of Medicine, Kagawa University, Kagawa, Japan; 3 Department of Head and Neck Surgery, International Medical Center, Saitama Medical University, Saitama, Japan; 4 Department of Head and Neck Surgery, Osaka International Cancer Institute, Osaka Prefectural Hospital Organization, Osaka, Japan; 5 Department of Otolaryngology-Head and Neck Surgery, Tohoku University Graduate School of Medicine, Miyagi, Japan; 6 Department of Otolaryngology-Head and Neck Surgery, Hyogo Medical University, Hyogo, Japan; 7 Department of Otorhinolaryngology, Head and Neck Surgery, Faculty of Medicine, University of the Ryukyus, Okinawa, Japan; 8 Department of Otolaryngology, Head and Neck Surgery, Niigata University Graduate School of Medical and Dental Sciences, Niigata, Japan; 9 Department of Otorhinolaryngology Graduate School of Medical Sciences, Kyushu University, Fukuoka, Japan; 10 Department of Otorhinolaryngology—Head and Neck Surgery, Osaka Rosai Hospital, Osaka, Japan; 11 Department of Otorhinolaryngology Head and Neck Surgery, Wakayama Medical University, Wakayama, Japan; 12 Department of Otorhinolaryngology—Head and Neck Surgery, Medical Science, University of Fukui, Fukui, Japan; 13 Department of Otolaryngology-Head and Neck Surgery, Keiyukai Sapporo Hospital, Sapporo, Japan; 14 Department of Otorhinolaryngology—Head and Neck Surgery, Tokushima University Graduate School of Biomedical Sciences, Tokushima, Japan; 15 Department of Otolaryngology, Head and Neck Surgery, School of Medicine, Tokai University, Kanagawa, Japan; 16 Division of Otolaryngology—Head and Neck Surgery, School of Medicine, Kanazawa University, Ishikawa, Japan; 17 Department of Otolaryngology—Head and Neck Surgery, Kurume University School of Medicine, Fukuoka, Japan; 18 Department of Otolaryngology Head and Neck Surgery, Kyoto Prefectural University of Medicine, Kyoto, Japan; 19 Department of Head and Neck Surgery, National Cancer Center Hospital, Tokyo, Japan; 20 Department of Head and Neck Oncology and Innovative Treatment, Graduate School of Medicine, Kyoto University, Kyoto, Japan; 21 Osaka General Medical Center Department of Otorhinolaryngology—Head and Neck Surgery, Osaka, Japan; 22 Department of Biostatistics, Yamaguchi University Graduate School of Medicine, Ube, Japan

**Keywords:** Recurrent or metastatic squamous cell carcinoma of the head and neck, First-line treatment, Modified EXTREME, Modified TPEx

## Abstract

**Background:**

The EXTREME regimen is the standard first-line treatment for recurrent or metastatic squamous cell carcinoma of the head and neck (R/M HNSCC), but it is poorly tolerated in Asian patients. We aimed to compare the efficacy and safety of the modified EXTREME (mEXTREME) and modified TPEx (mTPEx) regimens in Japanese patients.

**Methods:**

This was a multicenter, randomized, exploratory study. The dose of chemotherapeutic agents was reduced by 25% for the mEXTREME regimen compared to the EXTREME regimen and by 20% for the mTPEX regimen compared to the TPEx regimen. Six and four cycles were repeated every 21 days for the mEXTREME and mTPEx regimens, respectively. In both regimens, weekly 250 mg/m^2^ cetuximab was continued as maintenance therapy in case of disease control.

**Results:**

Sixty-one patients were enrolled and assigned to the two treatment arms (30 to mEXTREME, 31 to mTPEx). Median PFS was 6.0 months and 5.3 months (p = 0.28), and median overall survival was 17.4 months and 18.7 months (p = 0.72) for the mEXTREME and mTPEx groups, respectively. Twenty-seven patients in the mEXTREME group and 29 patients in the mTPEx group had grade 3 or worse adverse events during chemotherapy (p = 0.61). Early tumor shrinkage was 20% in the mEXTREME group and 44% in the mTPEx group (p = 0.01).

**Conclusions:**

No significant differences in survival were observed between the two groups, with similar toxicity profiles. As early tumor shrinkage was favorable in the mTPEx group, the mTPEx regimen may be the first-line standard of care for Asian patients with R/M HNSCC, particularly those who are not candidates for up-front pembrolizumab due to CPS < 1 or the presence of immunologically related complications and those who need rapid tumor shrinkage to relieve tumor-related symptoms.

**Trial registration:**

UMIN000025436.

**Supplementary Information:**

The online version contains supplementary material available at 10.1007/s10147-026-02964-w.

## Introduction

The EXTREME regimen that consists of six cycles of cisplatin/fluorouracil/cetuximab followed by weekly cetuximab has been the standard first-line treatment for recurrent or metastatic squamous cell carcinoma of the head and neck (R/M HNSCC) in patients with no history of platinum use or at least 6 months after platinum use [1]. However, as Asian patients tolerate chemotherapeutic agents less well than Western patients [2], the EXTREME regimen is commonly used at reduced doses in real-world settings in Asia. Clinical trials have been conducted in Asia to evaluate the efficacy of the less toxic modified EXTREME (mEXTREME) regimen with the cisplatin/fluorouracil doses reduced by 25% and the cetuximab dose maintained. Median overall survival (OS) was 11.1–12.6 months [3, 4], which was comparable to that with the EXTREME regimen (10.1 months) [1].

The TPEx regimen consists of four cycles of docetaxel/cisplatin/cetuximab followed by biweekly cetuximab. Prophylactic use of granulocyte colony-stimulating factor (G-CSF) is necessary to prevent febrile neutropenia. A phase 2 study on the efficacy of the TPEx regimen in Europe revealed promising results with median OS of 14.0 months [5]. Owing to the poor tolerability of Asian patients to chemotherapeutic agents and the approved docetaxel dose in Japan for head and neck cancer being 60 mg/m^2^, we devised the modified TPEx (mTPEx) regimen with 20% reduction of cisplatin/docetaxel dose. Furthermore, as cetuximab maintenance therapy for the mTPEx regimen, we used weekly 250 mg/m^2^ instead of biweekly 500 mg/m^2^ because administration of 500 mg/m^2^ cetuximab is not allowed in Japan. We retrospectively analyzed Japanese patients receiving the mTPEx regimen and found median OS of 14.3 months [6].

We initiated a phase 2 study to determine which regimen, the mEXTREME or mTPEx, is more beneficial in Japanese patients. However, patient enrollment did not proceed as expected because many patients who had previously received cisplatin-based chemoradiotherapy developed renal dysfunction and did not meet the inclusion criteria. Furthermore, two years after patient enrollment began, the results of a phase 3 study (KEYNOTE-048) were reported; pembrolizumab alone or in combination with platinum and fluorouracil improved OS over the EXTREME regimen in patients with a programmed cell death-ligand 1 (PD-L1) combined positive score (CPS) of ≥ 1 [7, 8]. Thereafter, in the standard of care for patients with CPS ≥ 1, the EXTREME regimen was replaced by pembrolizumab-based regimen, which further hampered patient recruitment. As enrolling the required number of patients for a phase 2 setting became difficult, we switched the setting of this trial from a phase 2 study to an exploratory study with a reduced sample size.

## Patients and methods

### Study design and participants

This randomized, exploratory study was conducted at 21 medical centers in Japan. The inclusion criteria were (1) age ≥ 20 years; (2) pathologically confirmed recurrent or metastatic squamous cell carcinoma of the oral cavity, oropharynx, hypopharynx, larynx, or nasal cavity and paranasal sinus not curable by local therapy; (3) Eastern Cooperative Oncology Group (ECOG) performance status score of 0 or 1; (4) presence of at least one tumor lesion measurable as per Response Evaluation Criteria in Solid Tumors (RECIST) version 1.1; (5) p16 expression status for oropharyngeal cancers; (6) clearance of creatinine > 60 mL/min. The exclusion criteria were (1) previous chemotherapy administration except if administered as part of a multimodal treatment for interested cancer at least 6 months before enrollment in the study or as treatment for metachronous second cancer at least three years before enrollment in the study; (2) administered cisplatin at an accumulated dose of > 300 mg/m^2^; (3) administered treatment with cetuximab or murine-derived monoclonal antibodies (including chimeric antibodies) within the previous 6 months. The inclusion and exclusion criteria are further elaborated in the protocol (Supplementary Information).

### Randomization and masking

Patients were randomly assigned (1:1) to receive the mEXTREME or mTPEx regimen using the dynamic allocation method at the registration/data center. Randomization was stratified by minimization according to the ECOG performance status score (0 vs. 1), p16 status for oropharyngeal cancer (positive vs. negative), and history of chemotherapy. Neither physicians nor patients were blinded to the treatment groups.

### Procedures

The mEXTREME regimen consisted of an intravenous infusion of 75 mg/m^2^ cisplatin on day 1, 750 mg/m^2^/day fluorouracil on days 1–5, and cetuximab on days 1, 8, and 15 (400 mg/m^2^ on day 1 of cycle 1 and 250 mg/m^2^ weekly for subsequent administration). Six cycles were repeated every 21 days. In case of disease control, intravenous cetuximab 250 mg/m^2^ was administered every week as maintenance therapy until disease progression or unacceptable toxicity.

The mTPEx regimen consisted of an intravenous infusion of 60 mg/m^2^ docetaxel on day 1, 60 mg/m^2^ cisplatin on day 1, and cetuximab on days 1, 8, and 15 (400 mg/m^2^ on day 1 of cycle 1 and 250 mg/m^2^ weekly for subsequent administration). Pegfilgrastim was injected subcutaneously at a dose of 3.6 mg on day 2 or 3 of each cycle as primary prophylactic G-CSF administration. Four cycles were repeated every 21 days, followed by maintenance cetuximab as aforementioned. In both groups, maintenance cetuximab therapy was initiated even if toxicity prohibited the continuation of chemotherapy. Dose modifications based on toxicity and replacement of cisplatin with carboplatin are detailed in the protocol.

Adverse events were monitored weekly during chemotherapy and maintenance therapy. Adverse events were evaluated and graded according to the Japan Clinical Oncology Group (JCOG) version of the National Cancer Institute’s Common Terminology Criteria for Adverse Events (CTCAE) version 4.0 (CTCAE v4.0—JCOG). The tumor response was assessed using computed tomography (CT) or magnetic resonance imaging (MRI) every 2 months after the start of treatment.

### Outcomes

The primary endpoint was progression-free survival (PFS), defined as the interval between registration and disease progression or death from any cause, whichever occurred first. The secondary endpoints were OS, defined as the interval between registration and death from any cause, response rate, defined as the percentage of patients with best overall response (complete or partial response), and safety. Shortly before the study initiation, nivolumab, an immune checkpoint inhibitor was approved for platinum-resistant R/M HNSCC treatment in Japan. As nivolumab was likely to affect the secondary endpoint of OS, PFS2 was evaluated as a post-hoc endpoint. PFS2 events in patients administered second-line treatment were defined as disease progression or death from any cause after the second-line treatment, while PFS2 events in patients not administered second-line treatment were defined as PFS events. We also evaluated early tumor shrinkage (ETS) and depth of response (DpR) as post-hoc analyses. ETS was defined as the percentage reduction in tumor load assessed at the first imaging study after treatment initiation relative to the baseline tumor load, while DpR was defined as the percentage of minimal tumor load after treatment initiation as compared to the baseline tumor load [9, 10]. The tumor load was measured as the sum of the longest diameters of all target lesions.

### Statistical analysis

As this study was exploratory, the sample size was not calculated based on a statistical hypothesis. Overall, 60 patients (30 per group) were selected as the number of patients that could be enrolled during the study period. The estimation accuracy for 30 patients per group was evaluated. Median PFS with the EXTREME regimen in the phase 2 study in Japan was 4.1 months [95% confidence interval (CI), 4.0–5.5] [11], median PFS with the mEXTREME regimen in the phase 2 study in China was 6.6 months (95% CI, 5.1–7.7) [3], and median PFS with the mTPEx regimen in the retrospective study in Japan was 8.6 months (95% CI, 3.9–10.0) [6]. Based on these findings, we assumed that median PFS with the mEXTREME and mTPEx regimens would be 6.0 and 8.5 months, respectively. Under the assumption that survival time followed an exponential distribution and discontinuation was 5%, the expected 95% CI widths of median PFS were 6.5 and 9.3 months, with 80% probability of being ≤ 6.8 and ≤ 11.5, for the mEXTREME and mTPEx regimens, respectively. The expected hazard ratio was 0.73, with an 80% probability of being < 0.98. This study assessed the PFS of each regimen in an exploratory manner; if the difference between the two therapies is of some magnitude, the results may be helpful in considering the next trial.

Time-to-event endpoints (PFS and OS) were estimated using the Kaplan–Meier method. The 95% CIs were calculated using the Brookmeyer and Crowley method. Hazard ratios (HRs) and 95% CIs were estimated using the Cox proportional hazards model. Differences between the groups were evaluated using the log-rank test. The rate of best overall response was estimated as the number of patients with complete or partial response at any time during first-line treatment among all patients. Response rates and proportions of patients receiving chemotherapy or cetuximab were compared using Fisher’s exact test. ETS and DpR were compared using the Mann–Whitney test. Patient characteristics were compared using the chi-square test and Student's t-test for categorical and continuous variables, respectively.

The efficacy was analyzed in the full analysis set (FAS) and per-protocol set (PPS). The FAS represented patients administered at least one dose of chemotherapy or cetuximab with some data obtained, while the PPS represented the subpopulation of the FAS, the patients with evaluable efficacy and no serious deviations or violations from the protocol provisions, including the dosage and administration schedule. Safety was analyzed in all patients administered at least one dose of chemotherapy or cetuximab (safety analysis set, SAS). All *p*-values were two-sided at a significance level of 0.05. SAS (version 9.4) was used for all statistical analyses.

## Results

**Fig. 1:**
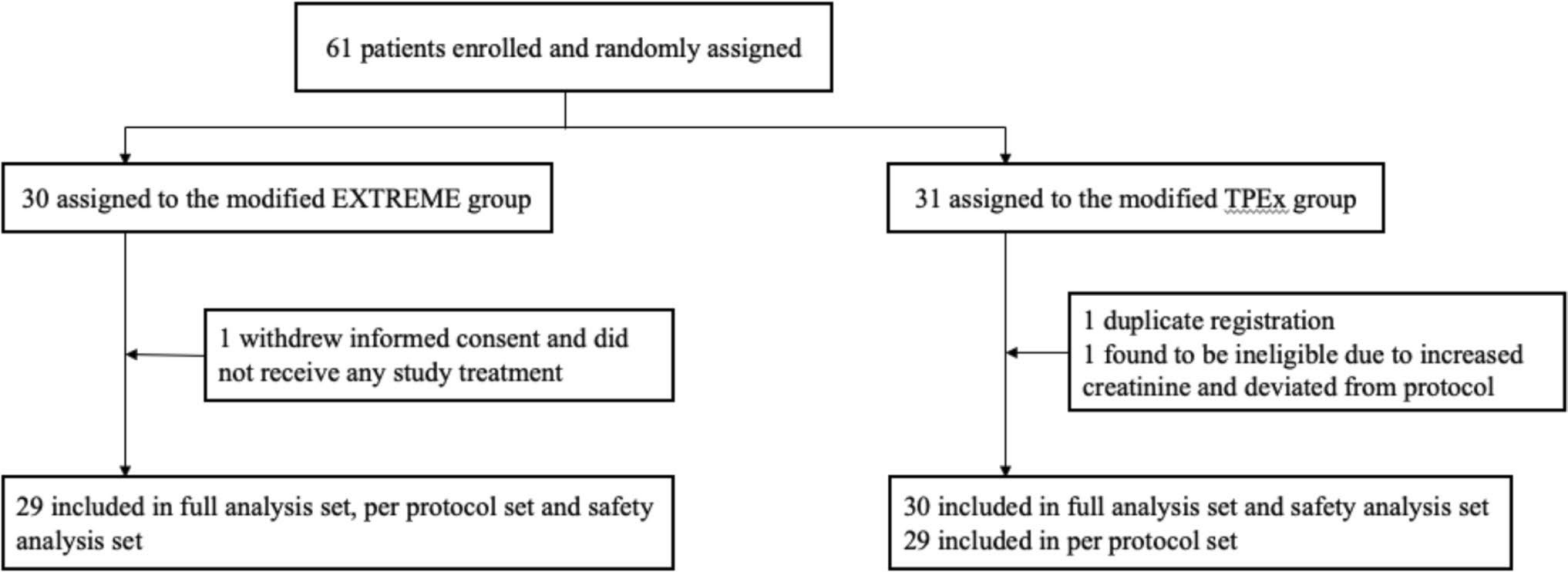
xxxx

Between October 2017 and July 2020, 61 patients were enrolled and randomly allocated to the two treatment groups (mEXTREME regimen, n = 30; mTPEx regimen, n = 31) (Fig. 1). One patient in the mEXTREME group was excluded from the FAS, PPS, and SAS as the patient withdrew informed consent. One patient in the mTPEx group was excluded from the FAS, PPS, and SAS as the patient was misregistered (duplicate registration). Another patient in the mTPEx group was excluded from the PPS as his renal function did not meet the inclusion criteria and he did not undergo imaging studies for 1 year after treatment discontinuation. The baseline patient characteristics are presented in Table 1.

**Table 1:** Patient characteristics

	mEXTREME (n = 29)	mTPEx (n = 30)	
Sex			
Male	27 (93%)	26 (87%)	*p* = 0.41
Female	2 (7%)	4 (13%)	
Age			
Median (range)	67 (54–82)	67 (43–80)	*p* = 0.92
ECOG performance status			
0	21 (72%)	22 (73%)	*p* = 0.94
1	8 (28%)	8 (27%)	
Primary tumor site			
Oral cavity	4 (14%)	10 (33%)	*p* = 0.06
Hypopharynx	9 (31%)	14 (47%)	
p16 + Oropharynx	5 (17%)	4 (13%)	
p16-Oropharynx	6 (21%)	1 (3%)	
Larynx	3 (10%)	1 (3%)	
Sinonasal	2 (7%)	0 (0%)	
Drinking			
Never	3 (10%)	3 (10%)	*p* = 0.97
Current	26 (90%)	27 (90%)	
Smoking			
Never	2 (7%)	4 (13%)	*p* = 0.02
Former	11 (38%)	20 (67%)	
Current	16 (55%)	6 (20%)	
Type of disease spread at inclusion			
Metastasis alone	10 (34%)	12 (40%)	*p* = 0.89
Locoregional relapse alone	9 (31%)	8 (27%)	
Metastasis and locoregional relapse	10 (34%)	10 (33%)	
Previous treatment			
No	9 (31%)	10 (33%)	*p* = 0.54
Yes	20 (69%)	20 (67%)	
Previous platinum agent administration			
No	18 (62%)	19 (63%)	*p* = 0.56
Yes	11 (38%)	11 (37%)	

*ECOG* Eastern Cooperative Oncology Group, *mEXTREME* modified cisplatin/fluorouracil/cetuximab, *mTPEx* modified docetaxel/cisplatin/cetuximab

Details of chemotherapy administration are shown in Table 2. The median duration of chemotherapy was 13 weeks (1–22) in the mEXTREME group and 9 weeks (3–13) in the mTPEx group. A significantly higher proportion of patients received the planned number of cycles in the mTPEx group than in the mEXTREME group (73% vs. 41%, *p* = 0.02). Chemotherapy doses were reduced less frequently in the mTPEx group than in the mEXTREME group. Cisplatin was replaced less frequently by carboplatin in the mTPEx group than in the mEXTREME group [10% (renal toxicities) vs. 24% (renal toxicities 10%, ototoxicities 14%)]. Maintenance cetuximab therapy was initiated more frequently in the mTPEx group than in the mEXTREME group (60% vs. 34%, *p* = 0.04). The median duration of maintenance was 16 weeks (1–105) in the mEXTREME group and 10 weeks (2–30) in the mTPEx group. Maintenance therapy is detailed in Online Resource 1.

**Table 2:** Treatment and response

	mEXTREME (n = 29)	mTPEx (n = 30)
Number of chemotherapy cycles received		
0	0	0
1	2 (7%)	0
2	4 (14%)	6 (20%)
3	5 (17%)	2 (7%)
4	4 (14%)	22 (73%)
5	2 (7%)	-
6	12 (41%)	-
Median of cycles	4	4
Median duration of chemotherapy (week)	13	9
Reason for chemotherapy discontinuation		
End of chemotherapy period	12 (41%)	22 (73%)
Disease progression	6 (21%)	6 (20%)
Adverse event	6 (21%)	2 (7%)
Patient refusal	5 (17%)	0
Maintenance therapy with cetuximab		
No	19 (66%)	12 (40%)
Yes	10 (34%)	18 (60%)
Median of cycles (range)	12 (1–84)	7 (2–28)
Median duration of administration (week)	16	10
Best overall response		
Complete response	2 (7%)	2 (7%)
Partial response	11 (38%)	18 (60%)
Stable disease	9 (31%)	4 (13%)
Progressive disease	5 (17%)	5 (17%)
Not evaluable	2 (7%)	1 (3%)
Response rate (95% confidence interval)	45% (26–64)	67% (47–83)

*mEXTREME* modified cisplatin/fluorouracil/cetuximab, *mTPEx* modified docetaxel/cisplatin/cetuximab

**Fig. 2:**
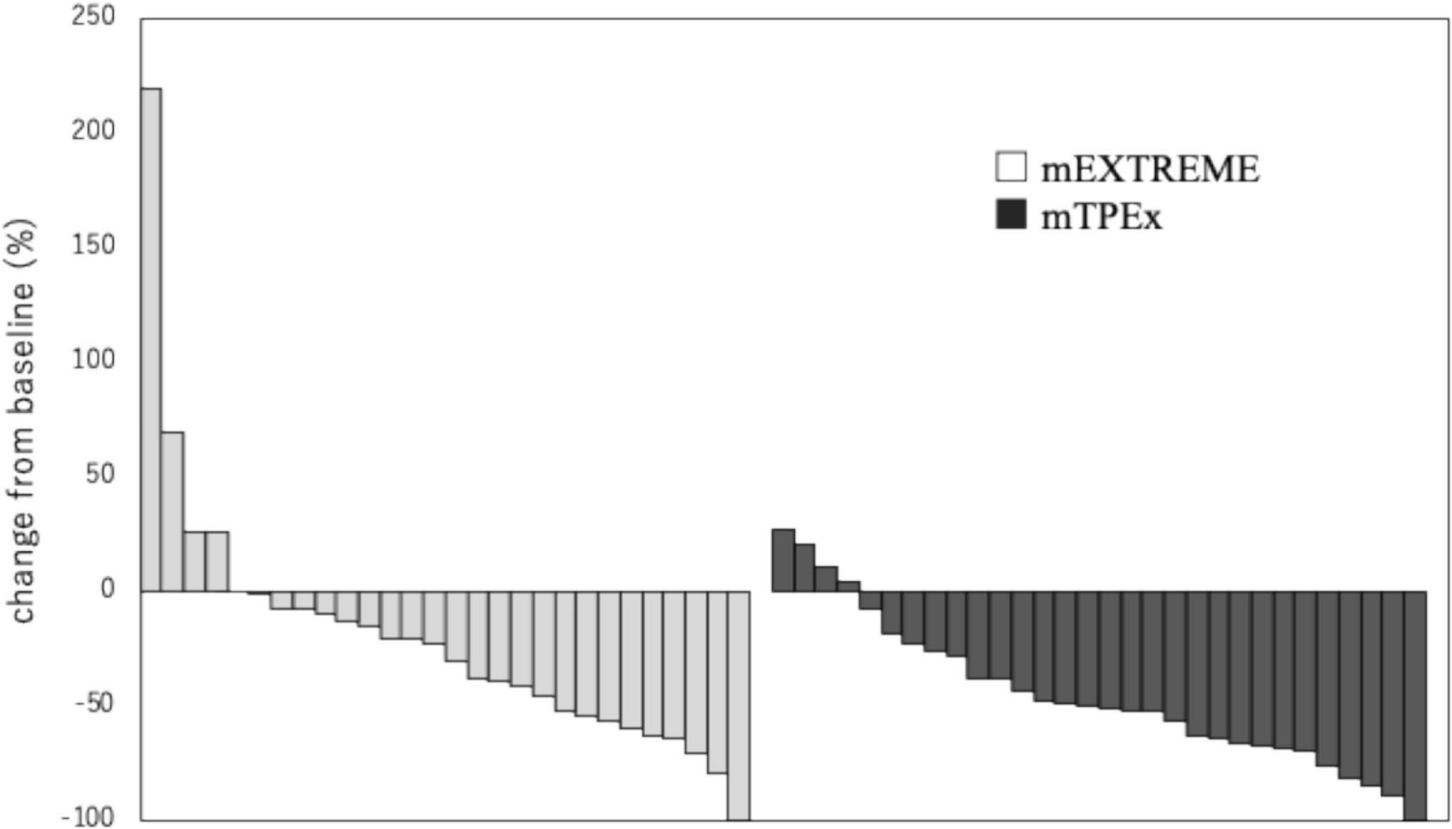
Waterfall plot for depth of response: best response by patient based on percentage change in summed longest diameter of target lesions from baseline

The best overall response was not significantly different between the groups (Table 2), with a response rate of 45% (95% CI, 26–64) in the mEXTREME group vs. 67% (95% CI, 47–83) in the mTPEx group (*p* = 0.12). Best overall response consists of complete response and partial response, but the tumor size reduction rate in partial response ranges from 30 to 99%, indicating some heterogeneity in best overall responses. Therefore, we evaluated the ETS and DpR for post-hoc analyses (Online Resource 2). Median ETS was 20% (95% CI, -10–32) in the mEXTREME group and 44% (95% CI, 28–49) in the mTPEx group; the difference was statistically significant (*p* = 0.01). Median DpR was better in the mTPEx group than in the mEXTREME group [51% (95% CI, 33–57) vs. 27% (95% CI, -2–44)], with no significant difference (*p* = 0.07). DpR is presented as a waterfall plot in Fig. 2.

**Fig. 3:**
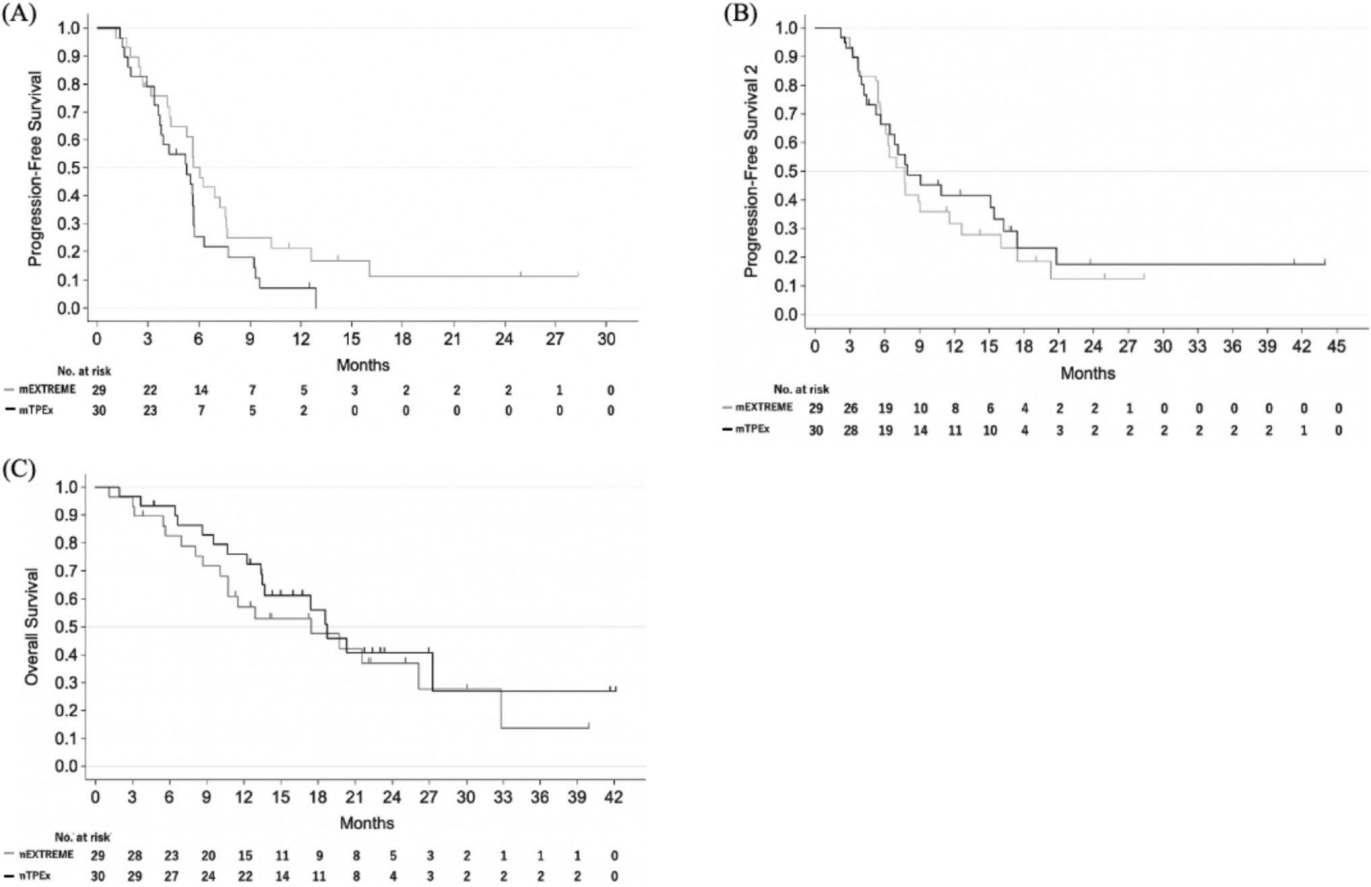
Kaplan–Meier estimates of survival: **A** Progression-free survival. **B** Progression-free survival 2. **C** Overall survival

The data were cut off on July 31, 2021. Median follow-up was 12.5 months (95% CI, 11.1–18.3) and 14.6 months (95% CI, 13.0–20.0) for the mEXTREME and the mTPEx groups, respectively. Overall, 51 disease progression events or deaths were observed across both groups (24 in the m EXTREME group and 27 in the mTPEx group). In the FAS, median PFS was 6.0 months (95% CI, 4.2–7.6) in the mEXTREME group and 5.3 months (95% CI, 3.6–5.7) in the mTPEx group (Fig. 3A). The stratified HR was 1.41 (95% CI, 0.76–2.62) with no significant difference (*p* = 0.28). PFS did not differ significantly in the PPS, either (*p* = 0.28). Fourteen patients (48%) in the mEXTREME group and 24 patients (80%) in the mTPEx group received second-line treatment (Online Resource 3). In the FAS, median PFS2 was 7.6 months (95% CI, 5.7–11.5) in the mEXTREME group and 7.9 months (95% CI, 5.6–16.3) in the mTPEx group (Fig. 3B). The stratified HR was 0.81 (95% CI, 0.4–1.6) with no significant difference (*p* = 0.53). The difference was not significant in the PPS, either (*p* = 0.68). At data cutoff, 34 patients had died (18 in the mEXTREME group and 16 in the mTPEx group). In the FAS, median OS was 17.4 months (95% CI, 10.1–26.0) in the mEXTREME group and 18.7 months (95% CI, 13.3–not reached) in the mTPEx group (Fig. 3C). The stratified HR was 0.88 (95% CI, 0.43–1.81) with no significant difference (*p* = 0.72). No significant difference was observed in the PPS, either (*p* = 0.28).

Table 3 lists observed adverse events of grade 3 or worse during chemotherapy. Twenty-seven (93%) of 29 patients in the mEXTREME group and 29 (97%) of 30 patients in the mTPEx group had at least one adverse event of grade 3 or worse (*p* = 0.61). No difference was observed between the groups in adverse events of grade 4 or worse, either (41% vs. 33%, *p* = 0.60). The most common hematological adverse event of grade 3 or worse was lymphopenia in both groups: 48% of the mEXTREME group and 63% of the mTPEx group. Neutropenia, grade 3 or worse, occurred in 24% of the mEXTREME group and 33% of the mTPEx group, and febrile neutropenia occurred in 10% and 13%, respectively. The most common nonhematological adverse event, grade 3 or worse, was hyponatremia, which occurred in 17% of the mEXTREME group and in 30% of the mTPEx group. Rash acneiform and anorexia occurred in 14% of the mEXTREME group and in 20% of the mTPEx group. Infusion reaction did not occur in any patient in the mEXTREME group, while 13% of the mTPEx group experienced it. In the mEXTREME group, one patient died of catheter-related infection, and one patient died by suicide (Online Resource 4).

**Table 3:** Adverse events during the chemotherapy phase

Assessments	Modified EXTREME (n = 29)										Modified TPEx (n = 30)									
	Grade 3		Grade 4		Grade 5		Grade 3 or worse		Any grade		Grade 3		Grade 4		Grade 5		Grade 3 or worse		Any grade	
Any types of adverse events	25	(86%)	11	(38%)	2	(7%)	27	(93%)	29	(100%)	28	(93%)	10	(33%)	0	(0%)	29	(97%)	30	(100%)
Hematologic toxicity																				
Leukopenia	7	(24%)	1	(3%)	0	(0%)	8	(28%)	21	(72%)	8	(27%)	2	(7%)	0	(0%)	10	(33%)	19	(63%)
Neutropenia	3	(10%)	4	(14%)	0	(0%)	7	(24%)	20	(69%)	5	(17%)	5	(17%)	0	(0%)	10	(33%)	16	(53%)
Febrile neutropenia	3	(10%)	0	(0%)	0	(0%)	3	(10%)	3	(10%)	4	(13%)	0	(0%)	0	(0%)	4	(13%)	4	(13%)
Lymphocytopenia	12	(41%)	2	(7%)	0	(0%)	14	(48%)	25	(86%)	15	(50%)	4	(13%)	0	(0%)	19	(63%)	28	(93%)
Thrombocytopenia	3	(10%)	1	(3%)	0	(0%)	4	(14%)	27	(93%)	2	(7%)	0	(0%)	0	(0%)	2	(7%)	23	(77%)
Anemia	6	(21%)	3	(10%)	0	(0%)	9	(31%)	29	(100%)	7	(23%)	1	(3%)	0	(0%)	8	(27%)	29	(97%)
Non hematologic toxicity																				
Rash acneiform	4	(14%)	0	(0%)	0	(0%)	4	(14%)	26	(90%)	6	(20%)	0	(0%)	0	(0%)	6	(20%)	28	(93%)
Diarrhea	4	(14%)	0	(0%)	0	(0%)	4	(14%)	10	(35%)	0	(0%)	0	(0%)	0	(0%)	0	(0%)	17	(57%)
Nausea	4	(14%)	0	(0%)	0	(0%)	4	(14%)	19	(66%)	1	(3%)	0	(0%)	0	(0%)	1	(3%)	20	(67%)
Anorexia	4	(14%)	0	(0%)	0	(0%)	4	(14%)	24	(83%)	6	(20%)	0	(0%)	0	(0%)	6	(20%)	26	(87%)
Dehydration	4	(14%)	0	(0%)	0	(0%)	4	(14%)	7	(24%)	1	(3%)	0	(0%)	0	(0%)	1	(3%)	8	(27%)
Hyponatremia	5	(17%)	0	(0%)	0	(0%)	5	(17%)	25	(86%)	8	(27%)	1	(3%)	0	(0%)	9	(30%)	27	(90%)
Hypokalemia	2	(7%)	0	(0%)	0	(0%)	2	(7%)	14	(48%)	5	(17%)	0	(0%)	0	(0%)	5	(17%)	17	(57%)
Infusion related reaction	0	(0%)	0	(0%)	0	(0%)	0	(0%)	0	(0%)	4	(13%)	0	(0%)	0	(0%)	4	(13%)	5	(17%)

Grade 3 or higher adverse events occurring in ≥ 10% in at least one group are shown. Patients who had different adverse events of different grades are counted in each grade for which they had at least one adverse event

## Discussion

This study revealed no significant differences between the mEXTREME and mTPEx regimens with respect to efficacy in terms of OS, PFS, and response rates in Japanese patients with R/M HNSCC. These results are in line with those of the GORTEC 2014–01 TPExtreme study [12]. The TPExtreme is a phase 2 study conducted in Europe that aimed to demonstrate the superiority of the TPEx regimen over the EXTREME regimen, with OS as the primary endpoint. The goal was not met as no difference was observed in OS between the groups.

In the TPExtreme study, the EXTREME group showed median PFS of 6.2 months and median OS of 13.4 months [12]. A phase 2 study evaluating the EXTREME regimen in Japanese patients showed similar results (median PFS of 4.1 months and median OS of 14.1 months) [11]. Compared to these results, PFS in this study (6.0 months in the mEXTREME group and 5.3 months in the mTPEx group) appears to be comparable and OS (17.4 months in the mEXTREME group and 18.7 months in the mTPEx group) improved. The improvement in OS may be attributed to the increased proportion of patients receiving immunotherapy as second-line treatment: 41–73% of patients in this study received immunotherapy, compared to 3–17% in the previous phase 2 studies [11, 12]. Asian patients treated with the mEXTREME regimen without second-line immunotherapy had median OS of 11.1–12.6 months [3, 4], further supporting the role of immunotherapy in improving OS in this study.

In the phase 2 trial evaluating the EXTREME regimen in Japanese patients, grade 4 or worse adverse events were observed in 64% of patients [11]. The most common grade 3 or worse adverse event was neutropenia, which occurred in 64% of patients [11]. In contrast, in this study, grade 4 or worse adverse events were observed in 41% of the mEXTREME group and 33% of the TPEx group, and grade 3 or worse neutropenia was observed in 24% of the mEXTREME group and 33% of the TPEx group. These results suggest that the mEXTREME and mTPEx regimens are less toxic and better tolerated than the EXTREME regimen. Infusion reaction was not observed in the mEXTREM group but was observed in 13% of the mTPEx group. This may be because docetaxel is included among chemotherapy drugs which may frequently cause infusion reaction, whereas fluorouracil is not [13].

Post-hoc analysis of the ETS revealed that tumor shrinkage was significantly faster in the mTPEx group than in the mEXTREME group. Thus, the mTPEx regimen may be preferable to the mEXTREME regimen when rapid relief from tumor-induced symptoms is required in case of CPS < 1. Even when CPS ≥ 1 indicates up-front pembrolizumab, if tumor-related symptoms have already appeared or are anticipated, rapid tumor shrinkage with mTPEx followed by sequential administration of pembrolizumab may be a viable treatment option. Moreover, by replacing fluorouracil with docetaxel, the mTPEX regimen has several other advantages over the mEXTREME regimen, including a shorter treatment duration (4 cycles vs. 6 cycles), easier delivery, and fewer contraindications.

This study had some limitations. Subgroup analysis was not performed owing to the small sample size. The small sample size also led to the wide 95% confidence intervals for the hazard ratios of PFS and OS. Data on CPS was lacking. Quality of life (QOL) assessment was planned but was not possible due to deficiencies in the collection of the QOL questionnaire. Hearing tests had been recommended to assess cisplatin ototoxicity, but they were rarely performed due to their inconvenience. Consequently, other QOL assessments were also underreported.

In conclusion, the mEXTREME and mTPEx groups showed no difference in efficacy and safety, while tumor shrinkage was faster in the mTPEx group than in the mEXTREME group. The mTPEx regimen might be the first-line standard of care for Asian patients with R/M HNSCC, particularly those who are not candidates for up-front pembrolizumab due to CPS < 1 or presence of immunologically related complications and those who need rapid tumor shrinkage to relieve tumor-related symptoms.

## Supplementary Information

Below is the link to the electronic supplementary material.

Supplementary file1 (PDF 379 KB)

## Data Availability

The data are available upon reasonable request. A proposal with detailed description of study objectives and statistical analysis plan will be needed for evaluation of the reasonability of requests. Additional materials might also be required during the process of evaluation. Deidentified participant data will be provided after approval from the corresponding author and study center.

## Declarations

### Trial registration

This study was registered at the University Hospital Medical Information Network (UMIN) database (Registration No. UMIN000025436; http://www.umin.ac.jp/ctr/) on December 27, 2016 and at the Japan Registry of Clinical Trials (jRCT) (Registration No. jRCTs071180009; https://jrct.mhlw.go.jp/) on November 29, 2018.

## References

[1] Vermorken JB, Mesia R, Rivera F et al (2008) Platinum-based chemotherapy plus cetuximab in head and neck cancer. N Engl J Med 359:1116–1127. 10.1056/NEJMoa0802656 18784101 10.1056/NEJMoa0802656

[2] O’Donnell PH, Dolan ME (2009) Cancer pharmacoethnicity: ethnic differences in susceptibility to the effects of chemotherapy. Clin Cancer Res 15:4806–4814. 10.1158/1078-0432.CCR-09-0344 19622575 10.1158/1078-0432.CCR-09-0344 PMC2774468

[3] Guo Y, Shi M, Yang A et al (2015) Platinum-based chemotherapy plus cetuximab first-line for Asian patients with recurrent and/or metastatic squamous cell carcinoma of the head and neck: results of an open-label, single-arm, multicenter trial. Head Neck 37:1081–1087. 10.1002/hed.23707 24710768 10.1002/hed.23707

[4] Guo Y, Luo Y, Zhang Q et al (2021) First-line treatment with chemotherapy plus cetuximab in Chinese patients with recurrent and/or metastatic squamous cell carcinoma of the head and neck: efficacy and safety results of the randomised, phase III CHANGE-2 trial. Eur J Cancer 156:35–45. 10.1016/j.ejca.2021.06.039 34418665 10.1016/j.ejca.2021.06.039

[5] Guigay J, Fayette J, Dillies AF et al (2015) Cetuximab, docetaxel, and cisplatin as first-line treatment in patients with recurrent or metastatic head and neck squamous cell carcinoma: a multicenter, phase II GORTEC study. Ann Oncol 26:1941–1947. 10.1093/annonc/mdv268 26109631 10.1093/annonc/mdv268

[6] Suzuki M, Takenaka Y, Kishikawa T et al (2021) Modified TPEx as first-line treatment for recurrent and/or metastatic head and neck cancer. Anticancer Res 41:2045–2051. 10.21873/anticanres.14973 33813412 10.21873/anticanres.14973

[7] Burtness B, Harrington KJ, Greil R et al (2019) Pembrolizumab alone or with chemotherapy versus cetuximab with chemotherapy for recurrent or metastatic squamous cell carcinoma of the head and neck (KEYNOTE-048): a randomised, open-label, phase 3 study. Lancet 394:1915–1928. 10.1016/S0140-6736(19)32591-7 31679945 10.1016/S0140-6736(19)32591-7

[8] Harrington KJ, Burtness B, Greil R et al (2023) Pembrolizumab with or without chemotherapy in recurrent or metastatic head and neck squamous cell carcinoma: updated results of the phase III KEYNOTE-048 study. J Clin Oncol 41:790–802. 10.1200/JCO.21.02508 36219809 10.1200/JCO.21.02508 PMC9902012

[9] Heinemann V, Stintzing S, Modest DP et al (2015) Early tumour shrinkage (ETS) and depth of response (DpR) in the treatment of patients with metastatic colorectal cancer (mCRC). Eur J Cancer 51:1927–1936. 10.1016/j.ejca.2015.06.116 26188850 10.1016/j.ejca.2015.06.116

[10] Xie X, Li X, Yao W (2021) A narrative review: depth of response as a predictor of the long-term outcomes for solid tumors. Transl Cancer Res 10:1119–1130. 10.21037/tcr-20-2547 35116438 10.21037/tcr-20-2547 PMC8797946

[11] Yoshino T, Hasegawa Y, Takahashi S et al (2013) Platinum-based chemotherapy plus cetuximab for the first-line treatment of Japanese patients with recurrent and/or metastatic squamous cell carcinoma of the head and neck: results of a phase II trial. Jpn J Clin Oncol 43:524–531. 10.1093/jjco/hyt034 23479384 10.1093/jjco/hyt034 PMC3638634

[12] Guigay J, Aupérin A, Fayette J et al (2021) Cetuximab, docetaxel, and cisplatin versus platinum, fluorouracil, and cetuximab as first-line treatment in patients with recurrent or metastatic head and neck squamous-cell carcinoma (GORTEC 2014-01 TPExtreme): a multicentre, open-label, randomised, phase 2 trial. Lancet Oncol 22:463–475. 10.1016/S1470-2045(20)30755-5 33684370 10.1016/S1470-2045(20)30755-5

[13] Roselló S, Blasco I, García Fabregat L et al (2017) Management of infusion reactions to systemic anticancer therapy: ESMO clinical practice guidelines. Ann Oncol 28(suppl_4):iv100–iv118. 10.1093/annonc/mdx216 28881914 10.1093/annonc/mdx216

