# Supplementary material for: Modified EXTREME regimen versus modified TPEx regimen as first-line treatment for recurrent or metastatic head and neck squamous cell carcinoma: a multicenter, open-label, randomized, exploratory study (TEMPER study)
Source: Int J Clin Oncol. 2026 Feb 9;31(4):611–20. doi: 10.1007/s10147-026-02964-w (PMC13018074; doi:10.1007/s10147-026-02964-w)
Supplement: Supplementary file 1 — Supplementary file1 (PDF 379 KB) [file 10147_2026_2964_MOESM1_ESM.pdf]

Online resource 1. Maintenance phase description.

	mEXTREME (n=29)	mTPEx (n=30)
Maintenance therapy with cetuximab		
No	19 (66%)	12 (40%)
Yes	10 (34%)	18 (60%)
Number of cetuximab administration during maintenance phase*		
Median (range)	12 (1-84)	7 (2-28)
Total dose of cetuximab received during maintenance phase (mg/m^2)*		
Median (range)	2675 (250 - 15500)	1750 (400 – 7000)
Reason for discontinuation of maintenance*		
Tumor progression	5 (50%)	16 (89%)
Patient refusal	1 (10%)	2 (11%)
Pneumonia	1 (10%)	0
On going	3 (30%)	0
Duration of maintenance phase*		
Median (in weeks) (range)	16 (1 - 105)	10 (2 - 30)

* In 28 patients who have finished maintenance phase, 10 in the mEXTREME group, 18 in the mTPEX group.

Online resource 2. ETS and DpR.

		modified EXTREME (n=28)	modified TPEx (n=30)	
Early tumor shrinkage	median (95% CI)	20% (-10 – 32)	44% (28 – 49)	*p* = 0.01
Depth of response	median (95% CI)	27% (-2 – 44)	51% (33 – 57)	*p* = 0.07

ETS (early tumor shrinkage), decrease in tumor burden measured on first imaging; DpR (depth of response), the smallest observed volume compared with the baseline.

Online resource 3. Second-line treatment.

	modified EXTREME (n=29)	modified TPEx (n=30)	
With second-line treatment			
Immunotherapy	12 (41%)	19 (63%)	
Immuno-chemotherapy	0	3 (10%)	
Other chemotherapy	2 (7%)	2 (7%)	
Without second-line treatment	15 (52%)	6 (20%)	$p = 0.02$

Online resource 4. Adverse events during the chemotherapy phase.

	modified EXTREME (n=29)						modified TPEx (n=30)					
Assessments	Grade 1-2	Grade 3	Grade 4	Grade 5	Grade 3 or worse	Any grade	Grade 1-2	Grade 3	Grade 4	Grade 5	Grade 3 or worse	Any grade
Hematologic toxicity												
Leukopenia	13 (45%)	7 (24%)	1 (3%)	0 (0%)	8 (28%)	21 (72%)	9 (30%)	8 (27%)	2 (7%)	0 (0%)	10 (33%)	19 (63%)
Neutropenia	13 (45%)	3 (10%)	4 (14%)	0 (0%)	7 (24%)	20 (69%)	6 (20%)	5 (17%)	5 (17%)	0 (0%)	10 (33%)	16 (53%)
Febrile neutropenia	0 (0%)	3 (10%)	0 (0%)	0 (0%)	3 (10%)	3 (10%)	0 (0%)	4 (13%)	0 (0%)	0 (0%)	4 (13%)	4 (13%)
Lymphocytopenia	11 (38%)	12 (41%)	2 (7%)	0 (0%)	14 (48%)	25 (86%)	9 (30%)	15 (50%)	4 (13%)	0 (0%)	19 (63%)	28 (93%)
Thrombocytopenia	23 (79%)	3 (10%)	1 (3%)	0 (0%)	4 (14%)	27 (93%)	21 (70%)	2 (7%)	0 (0%)	0 (0%)	2 (7%)	23 (77%)
Anemia	20 (69%)	6 (21%)	3 (10%)	0 (0%)	9 (31%)	29 (100%)	21 (70%)	7 (23%)	1 (3%)	0 (0%)	8 (27%)	29 (97%)
Cutaneous symptoms												
Rash acneiform	22 (76%)	4 (14%)	0 (0%)	0 (0%)	4 (14%)	26 (90%)	22 (76%)	6 (20%)	0 (0%)	0 (0%)	6 (20%)	28 (93%)
Rash maculo-papular	7 (24%)	2 (7%)	0 (0%)	0 (0%)	2 (7%)	9 (31%)	13 (43%)	0 (0%)	0 (0%)	0 (0%)	0 (0%)	13 (43%)
Alopecia	9 (31%)	0 (0%)	0 (0%)	0 (0%)	0 (0%)	9 (31%)	20 (67%)	0 (0%)	0 (0%)	0 (0%)	0 (0%)	20 (67%)
Paronychia	4 (14%)	1 (3%)	0 (0%)	0 (0%)	1 (3%)	5 (17%)	6 (20%)	2 (7%)	0 (0%)	0 (0%)	2 (7%)	8 (27%)
Gastrointestinal symptoms												
Diarrhea	6 (21%)	4 (14%)	0 (0%)	0 (0%)	4 (14%)	10 (35%)	17 (57%)	0 (0%)	0 (0%)	0 (0%)	0 (0%)	17 (57%)
Constipation	14 (48%)	0 (0%)	0 (0%)	0 (0%)	0 (0%)	14 (48%)	16 (53%)	0 (0%)	0 (0%)	0 (0%)	0 (0%)	16 (53%)
Nausea	15 (52%)	4 (14%)	0 (0%)	0 (0%)	4 (14%)	19 (66%)	19 (63%)	1 (3%)	0 (0%)	0 (0%)	1 (3%)	20 (67%)
Mucositis oral	15 (52%)	0 (0%)	0 (0%)	0 (0%)	0 (0%)	15 (52%)	14 (47%)	2 (7%)	0 (0%)	0 (0%)	2 (7%)	16 (53%)
Vomiting	4 (14%)	0 (0%)	0 (0%)	0 (0%)	0 (0%)	4 (14%)	10 (33%)	0 (0%)	0 (0%)	0 (0%)	0 (0%)	10 (33%)
Anorexia	20 (69%)	4 (14%)	0 (0%)	0 (0%)	4 (14%)	24 (83%)	20 (67%)	6 (20%)	0 (0%)	0 (0%)	6 (20%)	26 (87%)
Dehydration	3 (10%)	4 (14%)	0 (0%)	0 (0%)	4 (14%)	7 (24%)	7 (23%)	1 (3%)	0 (0%)	0 (0%)	1 (3%)	8 (27%)

	modified EXTREME (n=29)						modified TPEx (n=30)					
Assessments	Grade 1-2	Grade 3	Grade 4	Grade 5	Grade 3 or worse	Any grade	Grade 1-2	Grade 3	Grade 4	Grade 5	Grade 3 or worse	Any grade
Respiratory symptoms												
Dyspnea	4 (14%)	0 (0%)	0 (0%)	0 (0%)	0 (0%)	4 (14%)	2 (7%)	3 (10%)	0 (0%)	0 (0%)	3 (10%)	5 (17%)
Hypoxia	0 (0%)	1 (3%)	0 (0%)	0 (0%)	1 (3%)	1 (3%)	1 (3%)	2 (7%)	0 (0%)	0 (0%)	2 (7%)	3 (10%)
Pneumonitis	3 (10%)	0 (0%)	0 (0%)	0 (0%)	0 (0%)	3 (10%)	3 (10%)	1 (3%)	0 (0%)	0 (0%)	1 (3%)	4 (13%)
Aspiration	0 (0%)	0 (0%)	0 (0%)	0 (0%)	0 (0%)	0 (0%)	0 (0%)	0 (0%)	1 (3%)	0 (0%)	1 (3%)	1 (3%)
Cardiovascular system disorders												
Stroke	0 (0%)	0 (0%)	1 (3%)	0 (0%)	1 (3%)	1 (3%)	0 (0%)	0 (0%)	0 (0%)	0 (0%)	0 (0%)	0 (0%)
Peripheral ischemia	0 (0%)	0 (0%)	1 (3%)	0 (0%)	1 (3%)	1 (3%)	0 (0%)	0 (0%)	0 (0%)	0 (0%)	0 (0%)	0 (0%)
Acute limb ischemia	0 (0%)	0 (0%)	1 (3%)	0 (0%)	1 (3%)	1 (3%)	0 (0%)	0 (0%)	0 (0%)	0 (0%)	0 (0%)	0 (0%)
Acute coronary syndrome	0 (0%)	0 (0%)	0 (0%)	0 (0%)	0 (0%)	0 (0%)	0 (0%)	0 (0%)	1 (3%)	0 (0%)	1 (3%)	1 (3%)
Metabolism and nutrition disorders												
Hypoalbuminemia	28 (97%)	1 (3%)	0 (0%)	0 (0%)	1 (3%)	29 (100%)	28 (93%)	2 (7%)	0 (0%)	0 (0%)	2 (7%)	30 (100%)
AST increased	21 (72%)	0 (0%)	0 (0%)	0 (0%)	0 (0%)	21 (72%)	22 (76%)	1 (3%)	1 (3%)	0 (0%)	2 (7%)	24 (80%)
ALT increased	16 (55%)	1 (3%)	0 (0%)	0 (0%)	1 (3%)	17 (59%)	15 (50%)	2 (7%)	0 (0%)	0 (0%)	2 (7%)	17 (57%)
Creatinine increased	13 (45%)	0 (0%)	1 (3%)	0 (0%)	1 (3%)	14 (48%)	10 (33%)	0 (0%)	0 (0%)	0 (0%)	0 (0%)	10 (33%)
Hyponatremia	20 (69%)	5 (17%)	0 (0%)	0 (0%)	5 (17%)	25 (86%)	18 (60%)	8 (27%)	1 (3%)	0 (0%)	9 (30%)	27 (90%)
Hypokalemia	12 (41%)	2 (7%)	0 (0%)	0 (0%)	2 (7%)	14 (48%)	12 (40%)	5 (17%)	0 (0%)	0 (0%)	5 (17%)	17 (57%)
Hypocalcemia	20 (69%)	1 (3%)	1 (3%)	0 (0%)	2 (7%)	22 (76%)	13 (43%)	1 (3%)	0 (0%)	0 (0%)	1 (3%)	20 (67%)
Hypomagnesemia	23 (79%)	1 (3%)	1 (3%)	0 (0%)	2 (7%)	25 (86%)	27 (90%)	2 (7%)	0 (0%)	0 (0%)	2 (7%)	29 (97%)
Other events												
Catheter related infection	0 (0%)	0 (0%)	0 (0%)	1 (3%)	1 (3%)	1 (3%)	0 (0%)	1 (3%)	0 (0%)	0 (0%)	1 (3%)	1 (3%)
Suicide attempt	0 (0%)	0 (0%)	0 (0%)	1 (3%)	1 (3%)	1 (3%)	0 (0%)	0 (0%)	0 (0%)	0 (0%)	0 (0%)	0 (0%)
Infusion related reaction	0 (0%)	0 (0%)	0 (0%)	0 (0%)	0 (0%)	0 (0%)	1 (3%)	4 (13%)	0 (0%)	0 (0%)	4 (13%)	5 (17%)
Tinnitus	4 (14%)	0 (0%)	0 (0%)	0 (0%)	0 (0%)	4 (14%)	6 (20%)	0 (0%)	0 (0%)	0 (0%)	0 (0%)	6 (20%)

Any grade adverse events occurring in ≥ 10% in at least one group, grade 3 adverse events occurring in ≥ 5% in at least one group and any grade 4 or worse adverse events are shown.
